# Hybrid Assistive Limb Intervention in a Patient with Late Neurological Deterioration after Thoracic Myelopathy Surgery due to Ossification of the Ligamentum Flavum

**DOI:** 10.1155/2018/6171760

**Published:** 2018-02-08

**Authors:** Masakazu Taketomi, Yukiyo Shimizu, Hideki Kadone, Shigeki Kubota, Tetsuya Abe, Aiki Marushima, Tomoyuki Ueno, Ayumu Endo, Hiroaki Kawamoto, Akira Matsumura, Yoshiyuki Sankai, Yasushi Hada, Masashi Yamazaki

**Affiliations:** ^1^Department of Rehabilitation Medicine, University of Tsukuba Hospital, 2-1-1 Amakubo, Tsukuba, Ibaraki 305-8576, Japan; ^2^Center for Innovative Medicine and Engineering, University of Tsukuba Hospital, 2-1-1 Amakubo, Tsukuba, Ibaraki 305-8576, Japan; ^3^Division of Regenerative Medicine for Musculoskeletal System, Faculty of Medicine, University of Tsukuba, 1-1-1 Tennodai, Tsukuba, Ibaraki 305-8575, Japan; ^4^Department of Orthopaedic Surgery, Faculty of Medicine, University of Tsukuba, 1-1-1 Tennodai, Tsukuba, Ibaraki 305-8575, Japan; ^5^Department of Neurosurgery, Faculty of Medicine, University of Tsukuba, 1-1-1 Tennodai, Tsukuba, Ibaraki 305-8575, Japan; ^6^Faculty of Systems and Information Engineering, University of Tsukuba, 1-1-1 Tennodai, Tsukuba, Ibaraki 305-8573, Japan

## Abstract

**Purpose:**

We evaluated improvements in gait after using the Hybrid Assistive Limb (HAL®) exoskeleton robot in a patient with late-onset neurological deterioration of lower extremity function after undergoing thoracic spine surgery for a myelopathy due to ossification of the ligamentum flavum.

**Case Presentation:**

A 70-year-old man participated in ten 20 min sessions of HAL intervention, twice weekly for five weeks. The effects of each HAL session were evaluated based on changes in performance on the 10 m walk test (10 MWT), lower limb kinematics quantified from motion capture, and the activation ratio of the gastrocnemius, measured before and after the intervention. Muscle activity was recorded using surface electromyography and synchronized to measured kinematics. The HAL intervention improved gait speed and step length, with an increase in the hip flexion angle during the swing phase and a decrease in the activation ratio of the gastrocnemius. The modified Ashworth scale improved from 1+ to 1 and International Standards for Neurological and Functional Classification of Spinal Cord Injury motor scores from 34 to 49.

**Conclusion:**

Intervention using the HAL exoskeleton robot may be an effective method to improve functional ambulation in patients with chronic spinal disorders.

## 1. Introduction

**Figure 1:**
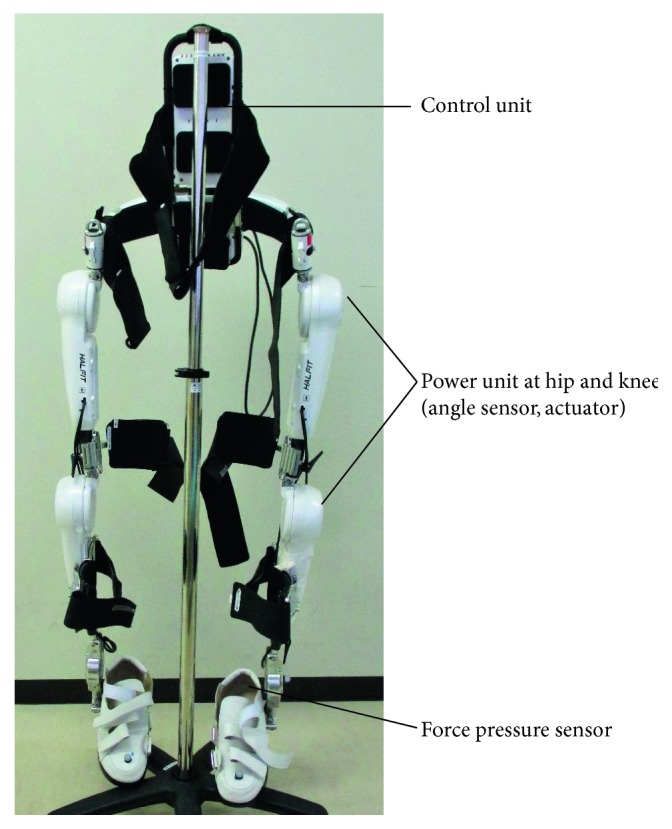
The exoskeleton Hybrid Assistive Limb (HAL), showing the power units of the hip and knee joints, bilaterally, which contain angular sensors, and the force-pressure sensors in the shoes.

Robotic devices have been shown to be beneficial during the physical rehabilitation of patients with neurological disorders, including those with spinal cord injuries [1, 2]. The Hybrid Assistive Limb (HAL) is an exoskeleton robot that assists the voluntary control of knee and hip joint motion bilaterally, by detecting signals from force-pressure sensors in shoes and weak bioelectric signals on the surface of the skin of associated muscles (Figure 1). Power units of the hip and knee joints of the exoskeleton suit include angular sensors and actuators for cybernic voluntary control (CVC) and cybernic autonomous control (CAC) [3]. HAL has unique operation system in using user's neuromuscular activities, compared to other rehabilitation robots such as Lokomat [4] (Hocoma, Switzerland), LOPES [5], or ReWalk (Robotics, Israel) [6], which are powered exoskeleton robots with angular sensors in the joints and pelvis and foot force-pressure sensors. Effective gait training outcomes using HAL have been reported in patients with chronic stroke [7–9] and spinal cord injury [9–12], as well as in patients following surgery for spinal myelopathy of the thoracic spine due to ossification of the posterior longitudinal ligament (OLF) [13–15]. We previously reported the effect for recovery of muscle activity during motion using HAL for chronic spinal cord injury [12, 16].

Gait dysfunction is the most important and common postoperative complication following surgery in patients with spinal myelopathy [13, 17, 18]; delayed-onset paralysis is a serious complication of the procedure. Lower limbs paralysis of the postoperative patients causes decreased walking distance, limitation of balance, and increased risk of falling. Such patients need rehabilitation to reduce the risk of falling, by an efficient use of their residual muscle activities, to maintain walking distance, and to keep their activities of daily living. In our case report, we describe improvement in gait obtained after a therapeutic intervention using the HAL robot for a patient who developed late-onset deterioration of neurological function after spinal surgery for OLF of the thoracic spine. Our intervention was conducted in accordance with the Declaration of Helsinki, with approval from the Ethics Committee of the Tsukuba University Faculty of Medicine (approval no.: H26-22). The patient provided informed consent for participation and publication, including the use of accompanying images.

## 2. Case Presentation

### 2.1. Patient, Clinical Findings, and Timeline

**Figure 2:**
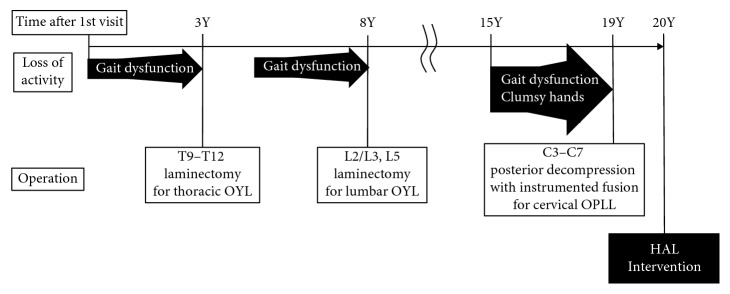
A summary of the progression of symptoms of the patient.

A 70-year-old man presented with progressive gait dysfunction following spinal surgery for a thoracic OLF. The progression of his gait dysfunction is summarized in Figure 2. The patient had undergone a T9–T12 laminectomy after being diagnosed with a thoracic OLF, 3 years after his first visit. Five years after the operation, he underwent a laminectomy of L2–L3 and L5 for a lumbar OLF, which was associated with lower limb pain and gait disturbance. Pain and gait function improved after the second spinal surgery. However, 7 years after the second operation, he experienced a renewed deterioration in his gait function, which was associated with limited balance ability, slower walking speed, and frequent falls, that restricted him to walking indoors only. He also experienced a loss of hand dexterity and sensation, with difficulty performing fine motor skills, such as fumbling with buttons. The patient was diagnosed with OLF again 11 years after the second operation and underwent posterior decompression with instrumented fusion of C3–C7. After the third surgery, hyperesthesia and motor impairment of his upper limbs improved, although the gait disturbances persisted. With 40 min of physical therapy, twice weekly, over several months following the third surgery, the patient regained the capacity to walk continuously for 10 min. However, his gait gradually deteriorated again, 1 year after his third surgery. He presented to our institution with difficulty walking indoors due to limited balance. Thinking of his situation as being chronic stage, we considered that motor learning to reduce the risk of falling was necessary rather than improvement of muscle strength. For this purpose, the decision to use the HAL for gait improvement was made, because of our experiences of the efficacy of HAL in motor learning [12, 16].

### 2.2. Diagnostic Assessment

**Figure 3:**
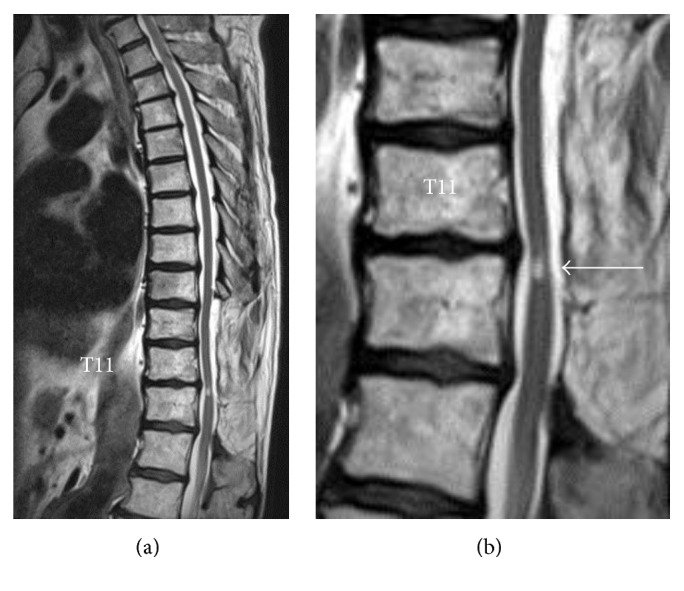
(a) Midsagittal T2-weighted magnetic resonance image of the thoracic spine, 2 years before the HAL intervention and 15 years after the T9–T12 laminectomy. (b) A high-power magnification view of (a), showing a high-intensity change persisting in the decompressed spinal cord at the T11-T12 level (arrow).

The T2-weighted magnetic resonance spinal image in Figure 3, obtained 2 years prior to the HAL intervention and 15 years after the T9–T12 laminectomy, shows a high-intensity change persisting in the spinal cord. From this finding, the patient was diagnosed with late-onset neurological deterioration due to spinal atrophy at the site of prior decompression of the thoracic spinal cord.

### 2.3. HAL Intervention

**Figure 4:**
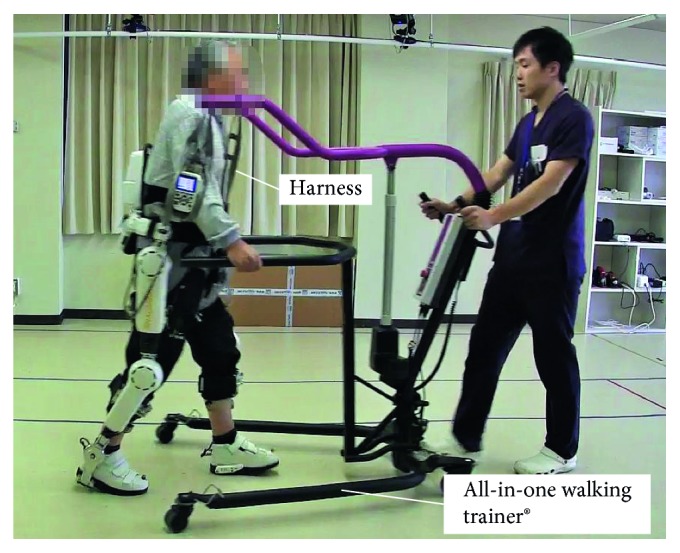
A photograph of the HAL exoskeleton, including the All-in-One Walking Trainer with the harness which was used during gait session to prevent a fall. The trainer provided consent for use of his image in this report.

The patient completed ten 20 min gait sessions using HAL during 5 consecutive weeks. Each session included 20-minute overground walk with HAL and 10 MWT without HAL before and after the session. All sessions were performed with a therapist, two assistants, and an engineer, under the supervision of a physician in case of emergency. The therapist and two assistants attached and detached the HAL exoskeleton suit, with the engineer implementing the gait analysis. For safety reasons, the All-in-One Walking Trainer (Ropox, Denmark), which includes a harness, was used for all sessions to prevent falls (Figure 4). In this case, the CVC mode was used as it supports a patient's voluntary motion by providing an assistive torque to each joint according to voluntary muscle activity. The CVC mode allows the operator to adjust the degree of physical support provided to the patient and to gradually reduce this support as session progresses.

### 2.4. Follow-Up

The 10 m walk test (10 MWT) was used to measure the primary outcome of the study and effectiveness of the intervention. The 10 MWT was performed without HAL or any assistance, but under supervision to ensure safety. The number of steps and time required to complete it were measured. Video recording and motion capture (MX with 16 T20S cameras; Vicon, UK) were used for the gait analysis, using the VICON Plug-in Gait marker placement for the lower limbs. Motion data were collected at 100 Hz, and the sagittal plane joint angles were computed using the Plug-in Gait model. Step length and cadence were also quantified from the kinematic data, using heel strike as the reference time point.

Activity of the gastrocnemius muscle was recorded, bilaterally, using a Trigno Lab wireless EMG system (Delsys, United States). The EMG data was collected at 2000 Hz in synchronization with the motion data and band-pass filtered at 30–400 Hz (using custom scripts, MATLAB, MathWorks, United States). The patient had a drop foot during swing phase, because of excessive and out-of-phase activation of the ankle plantar flexors. Therefore, the phase-dependent muscle activation of the plantar flexors was evaluated using the ratio of the integrated activity of the gastrocnemius during the swing phase to the activity of the muscle over the entire step cycle (swing and stance phase combined). The kinematic data for seven step cycles were extracted from each 10 MWT trial. For each of the extracted cycles, the maximum and minimum angles at the hip, knee, and ankle joints were extracted to calculate the joint range of motion. The kinematic profile of each lower limb joint for each extracted step cycle was normalized to the duration of the step cycle and averaged across cycles.

We also evaluated the modified Ashworth scale (mAs) of the gastrocnemius muscle [19] and the International Standards for Neurological and Functional Classification of Spinal Cord Injury (ISNCSCI) motor scores motor score as a measure of lower limb motor function.

All outcome variables were measured before and after the HAL intervention (PRE- and POST-10 sessions). Simple linear regression was used to examine the relationship between session number and changes in gait speed, cadence, and step length during the 10 MWT. All analyses were performed using JMP® 13.0.0 (SAS Institute Inc., Cary, NC, USA); a *p* value < 0.05 was considered significant.

### 2.5. Outcomes

**Figure 5:**
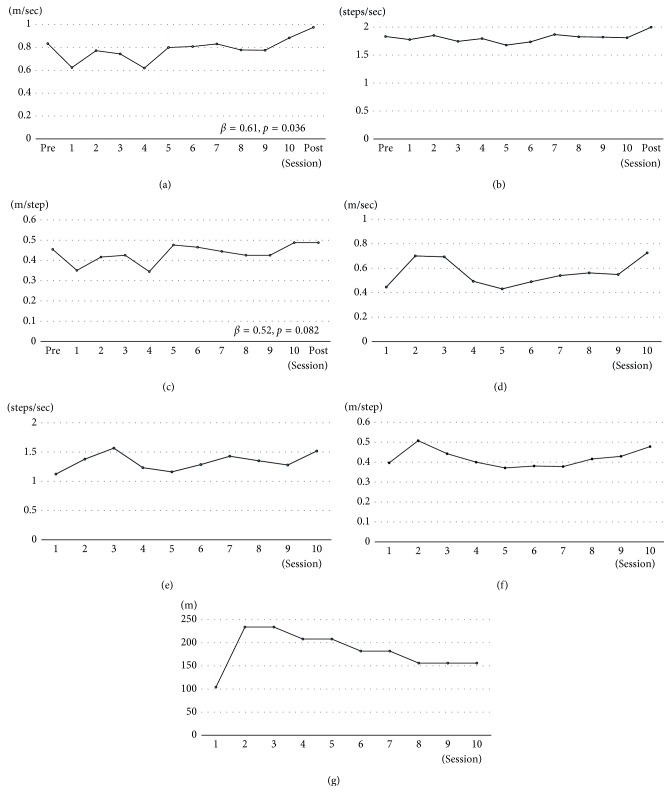
Graphs of the measured variables of the 10 MWT: (a) walking speed, (b) cadence, and (c) step length, measured without HAL, before each HAL session, and (d) walking speed, (e) cadence, (f) step length, and (g) walking distance, measured with the HAL, during each HAL session.

HAL intervention improved all measured features of gait performance during the 10 MWT, with the pre- to postintervention increases (Figures 5–5) in gait speed (0.83 m/s to 0.97 m/s; *β* = 0.61, *p*=0.036), cadence (1.83 steps/s to 2.00 steps/s), and step length (0.45 m to 0.48 m; *β* = 0.52, *p*=0.082). Figures 5–5 show those outcomes during HAL session.

**Figure 6:**
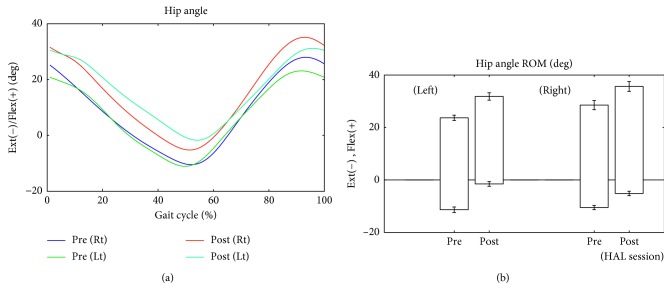
(a) Temporal profile of the angular position of the hip joint over the gait cycle, measured without the HAL, before and after the HAL intervention. (b) Range of motion of the hip over the gait cycle, measured without the HAL, before and after the HAL intervention. Error bars indicate the standard errors of the mean. Flex, flexion; Ext, extension; ROM, range of motion.

**Figure 7:**
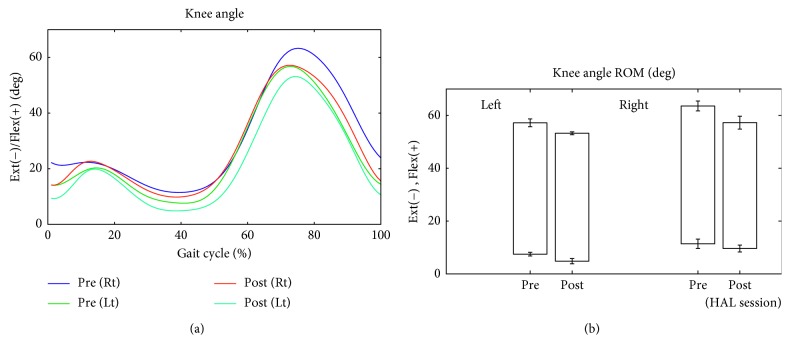
(a) Temporal profile of the angular position of the knee joint over the gait cycle, measured without the HAL, before and after the HAL intervention. (b) Range of motion of the knee over the gait cycle, measured without the HAL, before and after the HAL intervention. ROM, range of motion.

**Figure 8:**
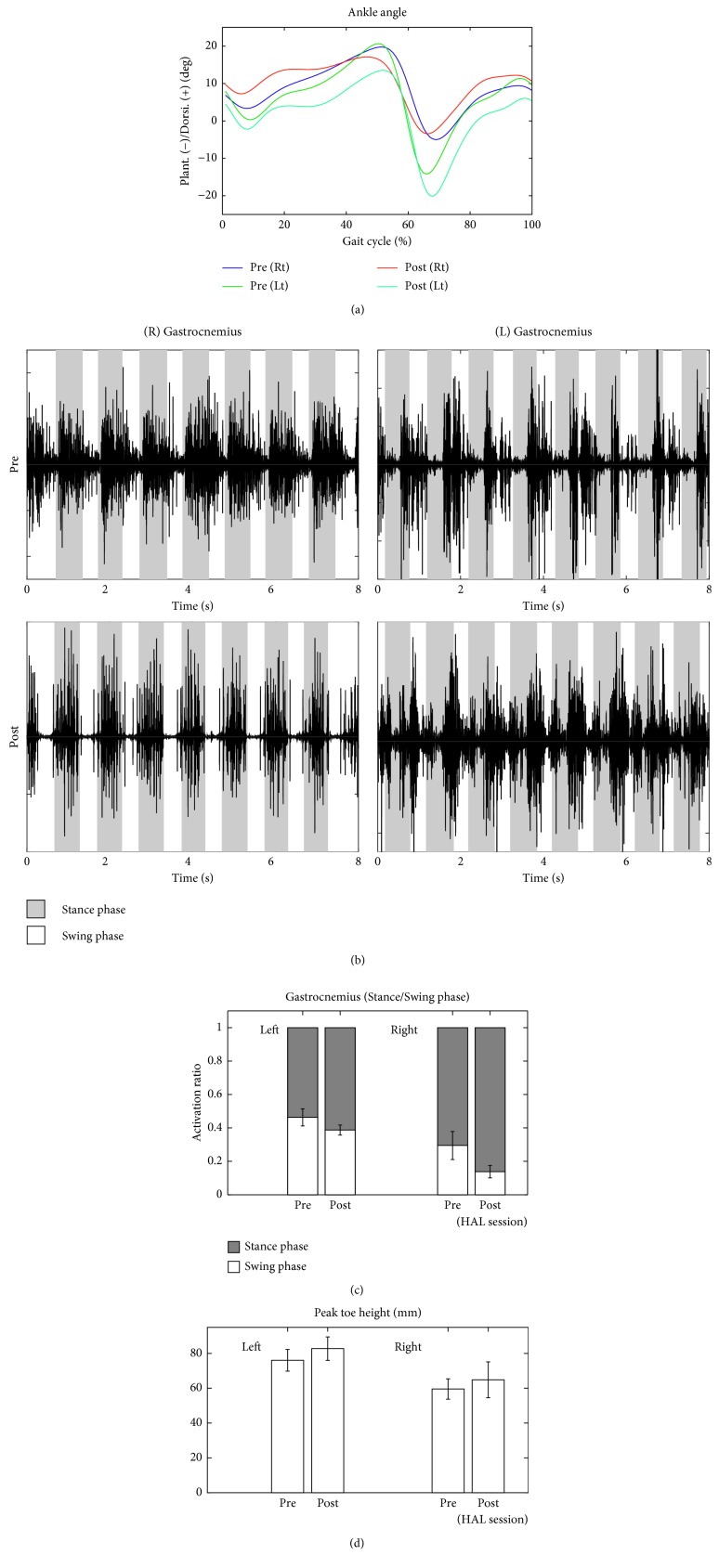
(a) Temporal profile of the angular position of the ankle joint over the gait cycle, measured without the HAL, before and after the HAL intervention. (b) Surface electromyography of the gastrocnemius muscles. (c) The muscle activation ratio of the gastrocnemius (swing phase to the total step cycle), before and after the HAL intervention. (d) Toe clearance, before and after the HAL intervention.

**Figure 9:**
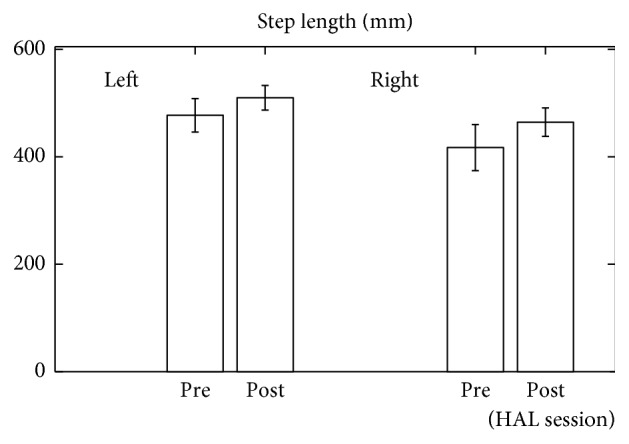
Step length before and after the HAL intervention.

The patient walked approximately 100 to 200 m during each session. Changes in lower limb kinematics are shown in Figures 6–9. After the HAL intervention, the mean ± SD maximum hip angle flexion during the swing phase increased from 23.4 ± 1.3° to 31.3 ± 1.0° on the left side and from 28.1 ± 1.6° to 35.3 ± 2.0° on the right side (Figure 6). Similarly, maximum flexion decreased at the knee from 57.2 ± 1.5° to 53.3 ± 0.6° on the left side and from 63.6 ± 1.9° to 57.3 ± 2.4° on the right side (Figure 7), with a further decrease in ankle plantar flexion, from 5.3 ± 1.8° to 3.6 ± 1.2° on the right side (Figure 8). Surface EMG profiles of the gastrocnemius are shown in Figure 8. The muscle activation ratio of the gastrocnemius decreased from 30 ± 10% to 14 ± 5% (Figure 8), while toe clearance increased from 59 ± 6 to 62 ± 7 mm (Figure 8). Step length increased from 477 ± 31 mm to 510 ± 23 mm on the left side and from 417 ± 43 to 464 ± 26 mm on the right side (Figure 9). The gastrocnemius mAs score improved from 1+ to 1. The lower limb ISNCSCI motor score improved from 34 to 49, with a noteworthy increase from 3 to 5 for left hip flexion and ankle plantar flexion, bilaterally. No adverse events associated with the HAL intervention were identified.

## 3. Discussion and Conclusions

In this case report, we describe improvement in the gait function of a 70-year-old patient with chronic spinal disorder following a gait intervention using the HAL exoskeleton robot. The delayed-onset paralysis in our patient was a possible symptom of spinal atrophy [20, 21], which can be associated with serious gait dysfunction that should not be ignored [13, 17, 18]. Surgical decompression can provide an effective treatment for spinal myelopathy. Typically, following decompression, neurological recovery of the upper limb function precedes recovery of lower limb function, with sphincter function being the last to recover and often being incomplete [22, 23]. Rehabilitation is usually needed to improve gait function postoperatively. In a systematic review of gait rehabilitation using the HAL, Wall et al. [11] reported the feasibility of the HAL exoskeleton suit to improve gait function in patients with lower extremity paresis in hospital and rehabilitation settings. Cruciger et al. [24] reported improved gait function using locomotion training with the HAL exoskeleton in patients with chronic neuropathic pain and those with chronic spinal cord injury.

HAL is a wearable robot suit to assist voluntary control of the knee and hip joint motion by detecting very weak bioelectric signals from associated musculature. However, other robotic devices, such as the Lokomat [4] and the LOPES [5], and powered exoskeleton devices, such as ReWalk [6], do not include sensors to detect the neuromuscular activation of wearers. Therefore, the HAL is unique in its use of innate neuromuscular control, compared with other rehabilitation robots, which can enhance motor learning [12, 16].

The HAL intervention we implemented improved specific components of gait (speed and step length), which subsequently improved ISNCSCI subscores of lower limb motor function, from 34 to 49 m. Moreover, the improvement in mAs score of the gastrocnemius indicates that the motion assist provided by the HAL during the sessions may be effective in reducing plantar flexor spasticity. Our previous study showed a similar reduction of spasticity after a HAL intervention in a patient with complete quadriplegia [25]. This decrease in spasticity may have contributed to improved muscle control and, therefore, strengthening of lower limb muscles. Improvements in the distance of independent walking and decrease in spasticity were considered as fundamental to the improvement in ISNCSCI scores. To our knowledge, this is the first report to have used gait analysis including kinematic and muscle activation data to quantify the effects of gait training using the HAL exoskeleton robot for a patient with a spinal cord disorder.

The greatest concern during locomotion for our patient was the risk of falling due to insufficient hip flexion and foot clearance over the swing phase of gait. After the HAL intervention, we identified a decrease in gastrocnemius activity during the swing phase, with an increase in activity over the stance phase. Plausible reason for the improved toe clearance might have been decreased gastrocnemius spasticity over the swing phase, which needs further investigation.

A previous study reported a significant decrease in ankle plantar flexor power in elderly individuals, compared with young adults [26, 27], with strengthening of the ankle plantar flexors being effective in improving stability during gait and preventing falls [28]. In our patient, a decrease in the range of plantar flexion of the right ankle during gait, compared with normal values, persisted even after the HAL intervention. However, manual muscle testing of the gastrocnemius improved from a score of “3/5” to “5/5”, bilaterally. We consider that this improvement in ankle plantar flexor strength would reduce the likelihood of falling. The increase in the flexion angle of the hip over the swing phase resulted in an increase in step length. This increase in the range of hip flexion, in combination with an increase in gastrocnemius activity during stance phase and a decrease during swing, as well as an increase in toe clearance, reduced the likelihood of falling, while increasing step length and improving gait function for daily locomotion. Therefore, our HAL intervention resulted in considerable functional improvement for our patient. This way, the gait improvement observed in the current case after HAL intervention indicates a new possibility of HAL: modification of gait to achieve improvement of daily activities in patients with limited balance ability because of neurological disorder, making comparison to effectiveness of gait training in the rather severe cases reported in previous reports [12–15, 25].

When evaluating the clinical applicability of our findings, it is important to note that this is a single case report and, therefore, a clinical trial is needed to evaluate the effectiveness of the HAL exoskeletal training in improving gait in patients with different conditions. This would be important to clearly establish guidelines for the use of HAL exoskeletal training in rehabilitation. Specifically, for patients with spinal cord injuries and disorders, case-control studies would be important to compare the effectiveness of the HAL intervention compared to conventional physical therapy or interventions using other robotic systems.

In conclusion, using a HAL-based intervention improved the gait of a patient presenting with late onset of neurological deterioration after surgery for thoracic myelopathy caused by OLF, including improvements of the ISNCSCI scores of lower limb motor function. Based on our experience, we propose that gait training using the HAL exoskeleton robot be used to provide a safe and feasible intervention to improve functional gait in patients with chronic spinal cord disorders.

## Abbreviations

HAL: Hybrid Assistive Limb
10 MWT: 10 m walk test
mAs: Modified Ashworth scale
ISNCSCI: The International Standards for Neurological and Functional Classification of Spinal Cord Injury
CVC: Cybernic voluntary control
CAC: Cybernic autonomous control
OLF: Posterior longitudinal ligament.
